# Lupeol Attenuates Palmitate-Induced Hypertrophy in 3T3-L1 Adipocytes

**DOI:** 10.3390/biom15010129

**Published:** 2025-01-15

**Authors:** Vaithinathan Selvaraju, Shivani R. Babu, Robert L. Judd, Thangiah Geetha

**Affiliations:** 1 Department of Nutritional Sciences, Auburn University, Auburn, AL 36849, USA; 2 Department of Anatomy, Physiology and Pharmacology, Auburn University, Auburn, AL 36849, USA

**Keywords:** lupeol, adipocyte hypertrophy, inflammation, obesity, adipokines

## Abstract

Obesity is characterized by the enlargement of adipose tissue due to an increased calorie intake exceeding the body’s energy expenditure. Changes in the size of adipose tissue can lead to harmful consequences, with excessive fat accumulation resulting in adipocyte hypertrophy and promoting metabolic dysfunction. These adiposity-associated pathologies can be influenced by dietary components and their potential health benefits. Lupeol, a pharmacologically active pentacyclic triterpenoid found in medicinal plants, vegetables, and fruits, has been shown to exhibit antioxidant and anti-inflammatory properties. This study investigated the role of lupeol on adipocyte hypertrophy by evaluating key adipogenic regulators in vitro. First, 3T3-L1 MBX mouse embryonic cells were differentiated into adipocytes and hypertrophy was induced using 500 µM palmitic acid. The treated adipocytes showed a significantly increased lipid droplet size, confirming adipocyte hypertrophy. Both adipocytes and hypertrophied adipocytes were then treated with or without 60 µM lupeol, following a dose-dependent study. Lipid droplet size was assessed and validated by Oil Red O staining. Western blot analysis was performed to measure the expression of adipogenic and inflammatory markers. Differentiated adipocytes showed increased fatty acid-binding protein 4 (FABP4) expression and Oil Red O staining, indicating an increased lipid content. Western blot analysis revealed that lupeol treatment reduced the expression of FABP4, peroxisome proliferator-activated receptor-γ (PPARγ), and adipokines. In conclusion, the results suggest that lupeol reverts the inflammatory and adipogenic markers that are enhanced in adipocyte hypertrophy. Through its anti-inflammatory effects, lupeol offers protective effects against adipocyte hypertrophy and contributes to reducing hypertrophic adiposity.

## 1. Introduction

Changing lifestyles can significantly impact both on human physical and mental health [1]. Obesity, often resulting from poor lifestyle habits, is strongly associated with various health issues, including type 2 diabetes, cardiovascular diseases, and cancer [2,3,4,5]. Obesity is a major public health problem in both developed and developing countries worldwide. According to the Centers for Disease Control and Prevention (CDC), the prevalence of obesity in U.S. has increased to 41.9% in 2017 to March 2020 [6]. Obesity-related medical costs are nearly $173 billion annually in the US [6,7]. The increasing prevalence of obesity highlights the urgent need to better understand its physiological mechanisms and related complications.

A positive energy balance between intake and expenditure increases weight gain and obesity. Nutrient intake variations can result in calorie surpluses, promoting the development of obesity. This occurs either by encouraging the growth of new fat cells from pre-existing ones (hyperplasia), leading to a metabolically healthy state, or by causing existing fat cells enlarge to store more lipids (hypertrophy), associated with a more unhealthy form of obesity [7]. The excess accumulation of fatty acid in the muscle and liver contributes to insulin resistance, a more damaging outcome of obesity than hyperplasia [8,9]. Hypertrophic adipocytes also experience hypoxia due to interactions with neighboring cells and increased extracellular matrix components, which leads to hypoxic stress and adipose tissue inflammation [10].

Dietary components may have a significant role in changing these adiposity-related pathologies by influencing adipose tissue growth, preadipocyte differentiation, lipolysis, and apoptosis. For example, quercetin, a flavonoid found in grapes, apples, blueberries, broccoli, onions, tea, and several other plants [11], has been shown to reduce pro-inflammatory signaling proteins and the expression of adipocytokines in 3T3-L1 adipocytes and fructose-fed rats [12]. Quercetin has shown to reduce adipocyte hypertrophy by enhancing adipogenesis activation in high-fat diet (HFD)-fed rats [13]. Similarly, phenolic compounds such as cyanidin-3-O-glycoside, protocatechuic acid, and ferulic acid present in purple maize extracts were found to reduce the inflammatory responses in TNF-α-induced adipocytes [14]. Further, protocatechuic acid has been found to decrease adipogenesis-induced inflammation and mitochondrial dysfunction in 3T3-L1 adipocytes by regulating AMPK pathways [15]. Paprika, commonly called sweet pepper, is rich in carotenoids, namely capsanthin and capsorubin [16]. These non-provitamin A carotenoids possess antioxidation, anti-inflammatory, and anti-cancer properties [17,18]. Paprika pigments have been reported to reduce the expression of obesity-related inflammatory markers, including interleukin-6 (IL-6), tumor necrosis factor-α (TNF-α), monocyte chemotactic protein-1 (MCP-1), and resistin in 3T3-L1 adipocytes [19].

**Figure 1:**
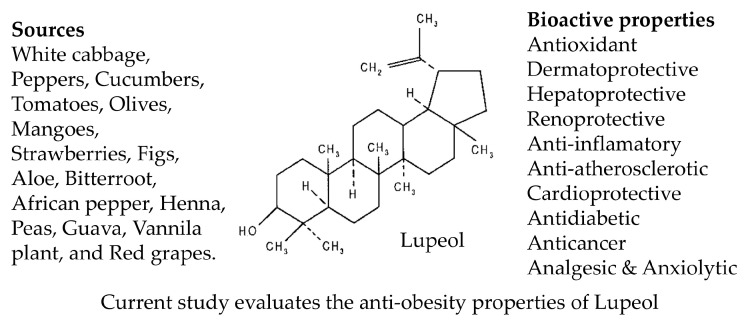
Lupeol structure, sources, and bioactive properties.

Lupeol, a plant-derived triterpenoid naturally found in various fruits, vegetables, and medicinal plants (Figure 1) such as white cabbage, peppers, cucumbers, tomatoes, mangoes, strawberries, and red grapes, has been shown to demonstrate significant antioxidant and anti-inflammatory properties [20,21]. Lupeol has shown protective effects against cardiac hypertrophy in mice by anti-inflammatory mechanisms involving the TLR-mediated PI3K pathway [22]. Another study reported that lupeol reduced inflammation by inhibiting IL-1 receptor-associated inflammatory signaling in mice [23]. Additionally, lupeol enhanced migration, wound healing, and contractile effects in human keratinocytes and fibroblasts through the PI3K/Akt and p38/ERK/MAPK pathways in a dose-dependent manner [24]. In the context of adipogenesis, lupeol regulates cell cycle progression and enhances glucose uptake in adipocytes [25]. Additionally, it inhibited inflammatory mediators linked to macrophage regulation and function in rats with diet-induced metabolic syndrome [26]. Lupeol has also been shown to prevent the differentiation of 3T3-L1 cells into mature adipocytes [27]. However, its effect on adipocyte hypertrophy, characterized by excessive lipid accumulation in adipocytes, remains unexplored. This study aims to evaluate the effects of lupeol on lipid accumulation in 3T3-L1 adipocytes treated with palmitic acid to induce hypertrophy. The differentiated adipocytes (control) and the hypertrophied adipocytes were treated with lupeol and the effects on lipid markers, adipokines, and inflammatory markers were evaluated.

## 2. Materials and Methods

### 2.1. Reagents and Antibodies

Dulbecco’s Modified Eagle medium (DMEM), fetal bovine serum (FBS), trypsin, EDTA, penicillin, and streptomycin were purchased from ATCC, Manassas, VA, USA. The antibodies FABP-4, adiponectin, PPARγ, MCP-1, p-GSK3β, GSK3β, p-NF-ĸB, NF-ĸB, p-JNK, JNK, p-Akt, Akt, -pERK, and ERK were purchased from Cell Signaling Technology (Danvers, MA, USA). GAPDH antibody, BODIPY staining, and ECL were obtained from Thermo Scientific, Waltham, MA, USA. Anti-rabbit IgG and anti-mouse IgG-HRP-linked secondary antibody were obtained from Enzo Life Sciences (Farmingdale, NY, USA). Lupeol, Oil Red O staining dye, insulin, sodium palmitate, and all other reagents were obtained from Sigma Aldrich, St. Louis, MO, USA.

### 2.2. 3T3-L1 Cell Culture and Differentiation to Adipocytes

A mouse embryonic fibroblast 3T3-L1 MBX clone (Catalog # CRL3242, Lot # 70023394, ATCC), derived from 3T3-L1 cells, was used in this study. The cells were cultured in Dulbecco’s Modified Eagle’s Medium (DMEM) supplemented with 10% fetal bovine serum (FBS) and penicillin/streptomycin. The 3T3-L1 cells were seeded into 6-well plates and differentiated using adipogenic differentiation media containing 0.5 mM 3-isobutyl-1-methylxanthine (IBMX), 1 μM dexamethasone, 10 μg/mL insulin, and 2 μM rosiglitazone for two days. On the third day, the cells were treated with insulin media (DMEM with 10 μg/mL insulin) for an additional two days. Subsequently, the cells were maintained in growth medium for ten days, during which the cells were fully differentiated into adipocytes.

### 2.3. Induction of Adipocyte Hypertrophy in 3T3-L1 MBX Cells

The palmitic acid (PA) solution for adipocytes was prepared by conjugating sodium palmitate with bovine serum albumin (BSA). Sodium palmitate (27.84 mg) was dissolved in 1 mL of 0.1 M NaOH and heated at 70–80 °C to create the PA stock solution. An intermittent stock solution was prepared by diluting the PA stock in DMEM medium containing 2% (wt/vol) free fatty acid (FFA)-BSA to a final concentration of 5 mM PA. Differentiated adipocytes were treated with a 500 µM PA working solution in DMEM medium for six days, with fresh PA solution replenished every 48 h. Before use, the PA solution in DMEM was filtered through a 0.22 µM filter. By the end of six days, the cells were hypertrophied and were ready for further treatment with lupeol.

Mouse embryonic fibroblast 3T3-L1 MBX cells were cultured in 6-well plates, differentiated into adipocytes (Day 10) using adipogenic differentiation media, and subsequently treated with a 500 µM PA working solution with DMEM medium for six days to induce hypertrophy in the adipocytes. Both the differentiated adipocytes (control) and the hypertrophied adipocytes were treated with lupeol for 48 h. Lupeol was dissolved in dimethyl sulfoxide (DMSO) as a stock solution and diluted to a working concentration of 60 µM. The dose was selected based on the preliminary results shown in Supplementary Figure S1.

### 2.4. Oil Red O Staining

Differentiated adipocytes were confirmed by Oil Red O staining. Briefly, the differentiated adipocytes were washed with PBS and fixed with 10% neutral buffered formalin (NBF) at room temperature for 1 h. After fixation, the cells were washed twice with distilled water and incubated with 60% isopropanol for 5 min. The isopropanol was replaced with the Oil Red O working solution and the cells were stained for 20 min at room temperature. Following staining, the cells were washed five times with distilled water to remove excess stain. The stained cells were imaged using a Revolve R4 Upright and Inverted Microscope. The same plates were read at 492 nm under a SpectraMax M2 plate reader and the results were quantified.

### 2.5. BODIPY Staining

Cells were fixed with 10% NBF and stained with BODIPY 493/503 (4,4-Difluoro-1,3,5,7,8-Pentamethyl-4-Bora-3a,4a-Diaza-s-Indacene) at a concentration 1 µg for 1 h at room temperature. After staining, the cells were washed three times with PBS and mounted using DAPI antifade ProLong Gold mounting medium. Images were captured using a Revolve R4 Upright and Inverted Microscope, Avantor ScienceCentral, Radnor, PA, USA.

### 2.6. Hypertrophied Adipocyte Size Measurement

The fat droplets in differentiated and hypertrophied adipocytes were imaged at 40× magnification using a Revolve R4 Upright and Inverted Microscope. Droplet sizes were analyzed using ImageJ software, version 1.53. The number of droplets with diameters < 10 µm and >10 µm diameter was quantified to validate the hypertrophy model.

### 2.7. Western Blot Analysis

Differentiated and hypertrophied adipocytes treated with lupeol were lysed with 2× Laemmli Sample Buffer. Western blotting (WB) was conducted to evaluate the adipogenic, inflammatory, survival pathway, and transcription factor proteins in the cell lysates. Equal amounts of undifferentiated and differentiated adipocyte cell lysates were resolved via SDS-PAGE and transferred to a PVDF membrane at a predetermined voltage (100 v) and time (1 h). The membranes were incubated with respective primary antibodies FABP-4 (Catalog # 2120, 1:1000 dilution); GSK3β (Catalog # 9315, 1:1000 dilution); JNK (Catalog # 9252, 1:1000 dilution); MCP-1 (Catalog # 2029, 1:1000 dilution); NFkB (Catalog # 8242, 1:1000 dilution); p-Akt (Catalog # 4060, 1:1000 dilution); p-ERK (Catalog # 9101, 1:1000 dilution); p-GSK3β (Catalog # 9336, 1:1000 dilution); p-JNK (Catalog # 4668, 1:1000 dilution); p-NFkB (Catalog # 3033, 1:1000 dilution); PPARγ (Catalog # 3435, 1:1000 dilution) overnight (all primary antibodies procured from Cell Signaling, Danvers, MA, USA). After primary incubation, the membranes were treated with HRP-conjugated secondary antibodies (1:5000 dilution) for 1 h. The membranes were thoroughly washed and developed using an enhanced chemiluminescence (ECL) detection reagent. To verify equal protein loading, the membranes were Western blotted with GAPDH as the loading control (Catalog # MA5-15738, Thermo Fisher Scientific, Waltham, MA, USA; 1:4000). The protein bands were quantified using ImageJ software.

### 2.8. Statistical Analysis

The data obtained from each analysis were organized in the Excel format. The data were verified for normality and the homoscedasticity assumption. Multiple group comparisons were analyzed using One-way Analysis of Variance (ANOVA), while two-group comparisons were performed using unpaired *t*-tests. Statistical analyses were conducted with GraphPad Prism 9 software. All values are given as the mean ± standard deviation (SD).

## 3. Results

**Figure 2:**
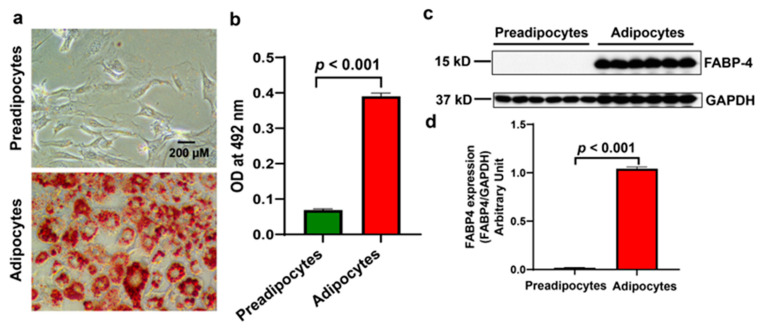
Differentiation of 3T3-L1 mouse embryonic fibroblasts to adipocytes. (**a**) Microscopic images showing Oil Red O staining of preadipocytes and adipocytes. (**b**) The graph shows the quantification of the staining measured at 492 nm absorbance using a spectrophotometer. (**c**) Preadipocytes and adipocyte lysates were Western blotted with FABP-4 and GAPDH antibodies. (**d**) The graph shows the quantification of FABP4 normalized with the GAPDH expression. Data shown as means ± SD, n = 3 per group.

First, the differentiation of the 3T3-L1 MBX cells to adipocytes was validated by Oil Red O staining and fatty acid-binding protein 4 (FABP4) Western blot analysis, as FABP4 is directly involved in lipid storage and lipolysis (Figure 2). The differentiated adipocytes showed significantly (*p* < 0.001) increased Oil Red O staining compared to preadipocytes (Figure 2a,b). The adipocytes also showed a significant increase in FABP4 expression by WB analysis compared to preadipocytes, as shown in Figure 2c,d. These results confirm the successful differentiation of 3T3-L1 cells into adipocytes.

**Figure 3:**
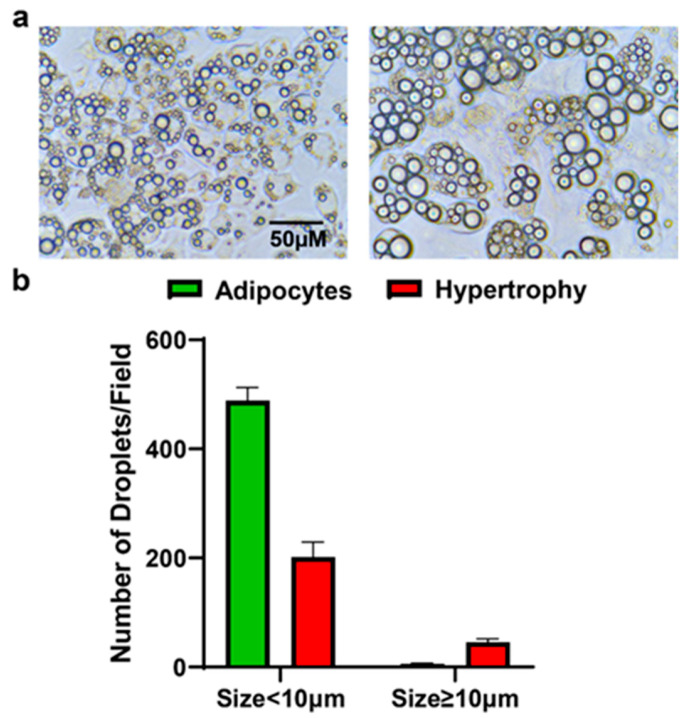
Lipid droplet size of adipocytes and hypertrophied adipocytes. (**a**) Representative microscopic images of lipid droplets in adipocytes and hypertrophied adipocytes. (**b**) The number of droplets with diameters < 10 µm and >10 µm diameter in adipocytes and hypertrophied adipocytes is shown as graph. Data shown as means ± SD, n = 3 per group.

After confirming the differentiation, hypertrophy was induced by treating the adipocytes with palmitic acid. The hypertrophied cells were compared to the differentiated adipocytes (control) based on the lipid droplet size. The microscopic images showed a marked increase in lipid droplet size, with droplets measuring 10 µM or more in the hypertrophied adipocytes compared to the control adipocytes (Figure 3). This result validates the adipocyte hypertrophy model induced by palmitic acid treatment.

**Figure 4:**
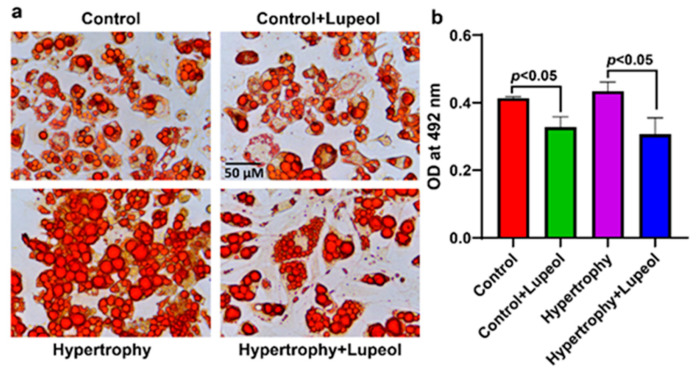
Effect of lupeol on control adipocytes and hypertrophied adipocytes by Oil Red O staining. (**a**) Representative microscopic images of Oil Red O staining of adipocytes and hypertrophied adipocytes treated with lupeol. (**b**) Quantification of staining of adipocytes and hypertrophied adipocytes treated with lupeol. Data shown as means ± SD, n = 3 per group.

**Figure 5:**
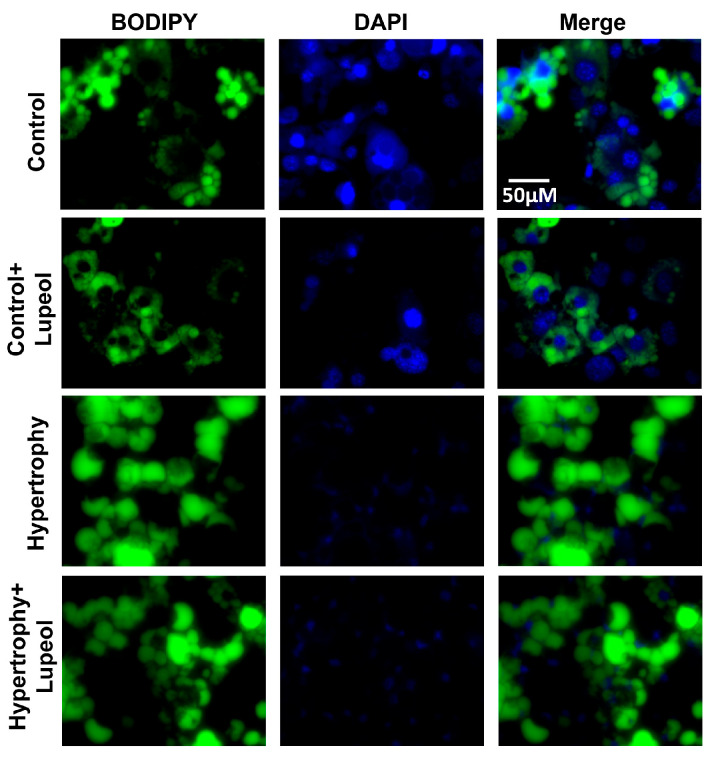
Representative microscopic images of BODIPY staining of lupeol-treated control adipocytes and hypertrophied adipocytes.

For dose-dependent studies, the adipocytes were treated with lupeol (20–60 μM) for 48 h. The lysates were Western blotted with the adipogenic marker FABP4. The expression of FABP4 was significantly decreased by 60 µM lupeol compared to the control adipocytes, as shown in Supplementary Figure S1. The effect of lupeol on the control adipocytes and hypertrophied adipocytes was evaluated using Oil Red O staining. The lupeol treatment significantly (*p* < 0.05) reduced the staining intensity in both adipocytes and hypertrophied adipocytes (Figure 4). The effect of lupeol was also determined by BODIPY 493/503 (4,4-difluoro-1,3,5,7,8-pentamethyl-4-bora-3a, 4a-diaza-s-indacene) staining. BODIPY specifically stained neutral lipids in the differentiated adipocytes, which also confirmed the adipocyte hypertrophy model. The images from the BODIPY staining reflect the lipids deposited in the droplets. After lupeol treatment, the lipids deposited in the droplets decreased in both differentiated and hypertrophied cells, as shown in the images in Figure 5.

**Figure 6:**
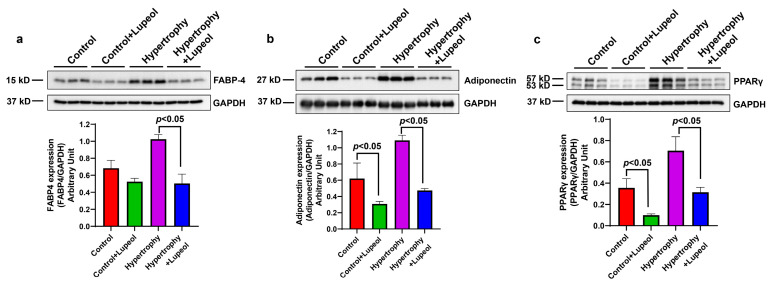
Effect of lupeol on control adipocytes and hypertrophied adipocytes on the expression of adipogenic proteins. Western blot analysis of lupeol-treated adipocytes and hypertrophied adipocytes lysates and quantification of (**a**) FABP4, (**b**) adiponectin, and (**c**) PPARγ normalized with GAPDH expression. Data shown as means ± SD, n = 3 per group.

Western blot analysis also provided some important insights into the effects of lupeol treatment on adipogenic-related molecules in both adipocytes and hypertrophied adipocytes. FABP4, an adipogenic marker and key regulator of lipogenesis and lipolysis, was significantly (*p* < 0.05) decreased after lupeol treatment in the hypertrophied adipocytes (Figure 6a). There were also significant (*p* < 0.05) reductions in the anti-inflammatory adipokine adiponectin after lupeol treatment in both adipocytes and hypertrophied adipocytes (Figure 6b). Furthermore, the expression of peroxisome proliferator-activated receptor-γ (PPARγ), a regulator of fatty acid metabolism, was also significantly (*p* < 0.05) decreased upon lupeol treatment (Figure 6c). These results suggest that lupeol may have potential as a therapeutic agent for mitigating obesity-related inflammation.

**Figure 7:**
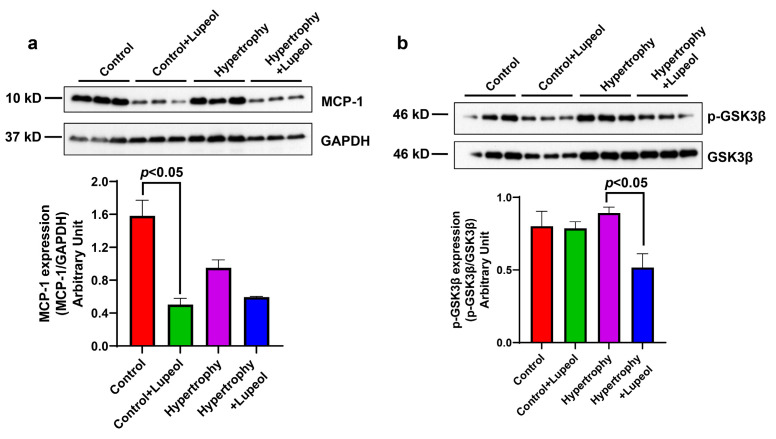
Effect of lupeol on control adipocytes and hypertrophied adipocytes on the expression of inflammatory proteins. Western blot analysis of lupeol-treated adipocytes and hypertrophied adipocytes lysates and quantification of (**a**) MCP-1 expression normalized with GAPDH and (**b**) p-GSK3β normalized with its non-phospho antibody. Data shown as means ± SD, n = 3 per group.

The effects of lupeol treatment on inflammatory markers were assessed by measuring the expression levels of monocyte chemoattractant protein-1 (MCP-1) and p-GSK3**β** by WB analysis. The expression of MCP-1 is reduced in adipocyte hypertrophy, but not significantly compared to the control adipocytes, which may be due to the fact that the inflammatory signals that stimulate MCP-1 production tend to decrease [28]. The lupeol treatment reduced the inflammatory marker MCP-1 in hypertrophied cells, with a significant reduction observed in control adipocytes (Figure 7a). Hoory markers were assessed by measuring the expression levels of monocyte chemoattractant protein-1 (MCP-1) an3β significantly (*p* < 0.05) in hypertrophied cells (Figure 7b). These results highlight the anti-inflammatory potential of lupeol, particularly in the context of hypertrophied adipocyte inflammation.

**Figure 8:**
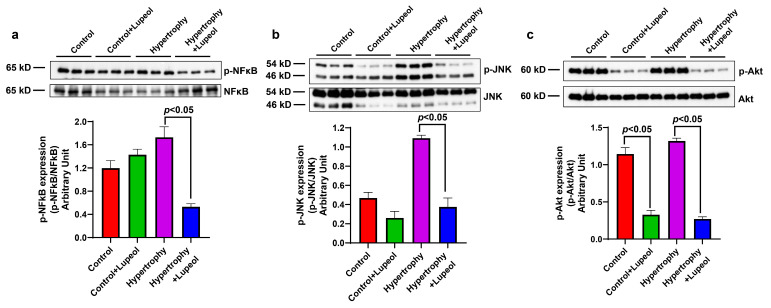
Effect of lupeol on control adipocytes and hypertrophied adipocytes on the expression of survival pathway proteins. Western blot analysis of lupeol-treated adipocytes and hypertrophied adipocytes lysates and quantification of (**a**) p-NF-ĸB, (**b**) p-JNK, and (**c**) p-Akt normalized with the corresponding non-phospho expression. Data shown as means ± SD, n = 3 per group.

Additionally, the effect of lupeol was also determined on the expression of survival pathway proteins. Inflammation induced by adipogenesis activates the N-terminal kinase (JNK) and nuclear factor kappa-light-chain-enhancer of activated B cell (NF-ĸB) signaling pathways in adipocytes and hypertrophied cells. Lupeol treatment significantly (*p* < 0.05) reduced the phosphorylation of NF-ĸB p65 and JNK in hypertrophied cells (Figure 8a,b). Furthermore, the treatment also significantly decreased the phosphorylation of Akt in both control and hypertrophied adipocytes (Figure 8c). These findings suggest that lupeol may modulate key survival signaling pathways involved in adipocyte inflammation and hypertrophy.

**Figure 9:**
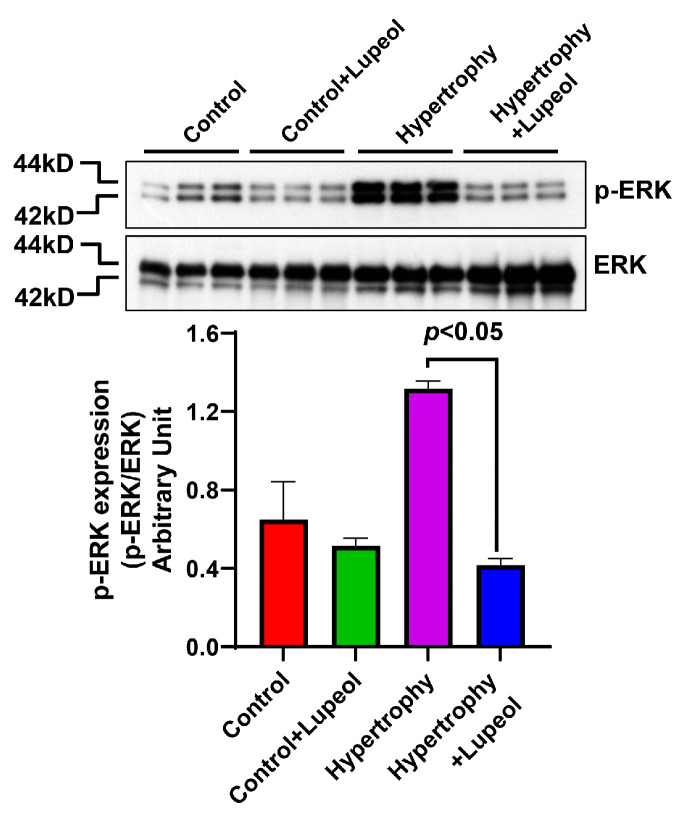
Effect of lupeol on control adipocytes and hypertrophied adipocytes on the expression of transcriptional factor proteins. Western blot analysis of lupeol-treated adipocytes and hypertrophied adipocytes lysates and quantification of p-ERK normalized with non-phospho ERK expression. Data shown as means ± SD, n = 3 per group.

In addition, the impact of lupeol treatment on ERK signaling was also evaluated. Western blot analysis of phosphorylated ERK (p-ERK) expression in the control and hypertrophied adipocytes revealed a significant (*p* < 0.05) decrease in p-ERK levels in hypertrophied cells treated with lupeol compared to those in the corresponding control group (Figure 9). This result supports the notion that lupeol treatment can mitigate adipocyte hypertrophy. Overall, these findings suggest that lupeol treatment can potentially reduce inflammation and adiposity in hypertrophied cells.

## 4. Discussion

Obesity, characterized by the excessive accumulation of body fat, can lead to various health complications. Current research efforts are focused on uncovering the mechanisms underlying obesity and developing targeted interventions to prevent and treat this condition. Studies have shown that obesity is closely linked to inflammation in adipose tissue [29]. Previous research has shown that cytokines and free fatty acids (FFAs) secreted by hypertrophic adipocytes induce macrophages to infiltrate adipose tissue, impairing the inflammatory response and contributing to metabolic dysfunction [30,31]. Understanding the properties of hypertrophied adipocytes that regulate insulin sensitivity and glucose metabolism has provided a deeper understanding of their role in maintaining insulin homeostasis.

Recent findings provide evidence for the use of plant-derived inhibitors of adipocyte differentiation as therapeutic strategies for managing obesity and reducing adipose tissue inflammation [22,30]. Our findings show that lupeol treatment reduces lipid droplet size and content in both differentiated and hypertrophied adipocytes, as observed through Oil Red O staining. PA-induced hypertrophy in 3T3-L1 adipocytes led to excessive lipid accumulation and the enlargement of lipid droplets, consistent with prior findings that PA causes DNA damage and lipid accumulation in adipocytes [32,33]. High consumption of PA is also associated with an increased expression of pro-inflammatory cytokines such as TNF-α, IL-6, and IL-1β [34].

Similar to previous research, this study found that PA-induced adipocyte hypertrophy is associated with an increased expression of PPARγ, a key regulator of fatty acid storage [26]. Lupeol treatment effectively diminished PPARγ levels, thereby reducing lipid accumulation. Lupeol has been found to reduce the expression of C/EBPα, PPARγ, and inflammatory cytokines in 3T3-L1 cells as well as animal models of obesity [13,35]. Adiponectin is found to be increased during early adipocyte hypertrophy and its expression may increase as a compensatory response to maintain metabolic balance [36]. We found that lupeol treatment significantly reduced the adiponectin expression in both differentiated adipocytes and hypertrophied adipocytes. By modulating adiponectin, lupeol impacts the metabolic balance and lipid regulation in hypertrophied adipocytes. While some studies suggest that adiponectin levels are negatively associated with obesity [37], others have reported an increased expression of adiponectin and PPARγ levels in adipocytes under certain conditions [38]. The role of adiponectin and PPARγ in adipocyte hypertrophy remains complex, given its crucial roles in regulating adipogenesis [39].

FABP-4 expression, which increases in hypertrophic adipocytes to manage excess lipid load, was also reduced following lupeol treatment. The activation of PPARγ also induces the expression of FABP-4 to promote lipid storage [40,41], but excessive FABP4 may exacerbate inflammation and metabolic dysfunction. FABPs act as important lipid chaperones and regulate lipid homeostasis [42,43]. The reduction in PPARγ and FABP4 indicates lupeol’s ability to counteract adipocyte hypertrophy by disrupting key adipogenic pathways. Lupeol treatment may reverse the increases of FABP-4, adiponectin, and PPARγ expression, highlighting its potential therapeutic benefits in managing hypertrophy and inflammation.

Our study also investigated the impact of lupeol on inflammatory markers, particularly MCP-1, and lupeol significantly reduced the expression of MCP-1 in hypertrophied cells. Previous studies have demonstrated that agents like pioglitazone improve metabolic health by increasing adiponectin and reducing MCP-1 secretion [44]. We further investigated the activation of PI3K-Akt, JNK, and NF-κB, which are survival pathway proteins involved in adipocyte inflammation and hypertrophy [45,46,47]. The activation of inflammatory signaling pathways, including NF-κB, JNK, and Akt, were significantly suppressed by lupeol treatment in hypertrophied adipocytes. This supports previous research showing that lupeol inhibits the NF-κB pathway, alleviating inflammation in macrophages and potentially offering therapeutic benefits for conditions such as cardiac remodeling and chronic inflammatory diseases [17,37].

In addition, lupeol treatment markedly reduced the activation of the pro-inflammatory kinase JNK, a critical mediator of chronic inflammation in hypertrophic and hypoxic adipocytes. Hypertrophied cells from high-fat diet (HFD)-fed rats have been shown to exhibit elevated levels of TLR-4, CD68, MCP-1, and JNK, driving the transcription of pro-inflammatory cytokines and perpetuating inflammation [12]. The expression of p-ERK was increased in adipocyte hypertrophy, but not significantly compared to control adipocytes. This might be due to the accumulation of excessive lipids that trigger the activation of the ERK signaling pathway. The ERK signaling pathway is involved in cell proliferation, differentiation, and metabolism [48]. The phosphorylation of ERK can also regulate the expression of PPARγ [49], as shown with the increase in the expression of PPARγ in adipocyte hypertrophy. ERK phosphorylation increases lipolysis, which in turn enhances the release and production of free fatty acids that cause the activation of NF-κB in adipocyte hypertrophy. The increased activity of ERK and NF-κB in adipocyte hypertrophy causes dysregulation in the expression of adipocytokines in adipocytes [48]. The ERK signaling pathway, known to regulate gene expression in hypertrophied adipocytes, was significantly modulated by lupeol, as evidenced by a reduced p-ERK/ERK ratio. This aligns with previous findings that drugs like metformin regulate gene expression in adipocytes through activating ERK signaling [38]. Lupeol reduces ERK phosphorylation, disrupting its role in adipocyte hypertrophy and inflammatory signaling. The modulation of ERK activity reduces lipid droplet size and inflammatory cytokine production. Lupeol reduces adipocyte hypertrophy and associated inflammation, providing a promising strategy for prevention and treatment of obesity.

While these findings are encouraging, this study has limitations. As a preliminary investigation, it primarily assessed signaling pathways without establishing causal relationships driving the observed effects of lupeol in obesity-related inflammation and adiposity. Further research is needed to elucidate the precise mechanisms underlying these effects and to validate the therapeutic potential of lupeol in clinical settings.

## 5. Conclusions

In conclusion, the results suggest that lupeol treatment effectively protects the palmitate-induced hypertrophy in 3T3-L1 adipocytes. Through its anti-inflammatory effects, lupeol attenuates adipocyte hypertrophy and contributes to reducing hypertrophic adiposity. These findings provide valuable insights into the potential role of lupeol as a therapeutic agent for preventing obesity and its related complications. Future research using in vivo experimental models is essential to elucidate the precise mechanisms underlying these effects and to establish causal relationships, paving the way for future targeted obesity interventions.

## Data Availability

The original contributions presented in the study are included in the article; further inquiries can be directed to the corresponding author.

## Supplementary Materials

The following supporting information can be downloaded at: https://www.mdpi.com/article/10.3390/biom15010129/s1, Figure S1: Dose-dependent effect of lupeol on the expression of FABP-4 by Western blot analysis. Original images of Figure 2c and Figure 6, Figure 7, Figure 8 and Figure 9 can be found in Supplementary materials.
