# Supplementary material for: Lupeol Attenuates Palmitate-Induced Hypertrophy in 3T3-L1 Adipocytes
Source: Biomolecules. 2025 Jan 15;15(1):129. doi: 10.3390/biom15010129 (PMC11763665; doi:10.3390/biom15010129)
Supplement: Supplementary file 1 [file biomolecules-15-00129-s001.zip › biomolecules-3376086-supplementary.pdf]

Supporting Information

Lupeol Attenuates Palmitate-Induced Hypertrophy in 3T3-L1 Adipocytes

Vaithinathan Selvaraju [1], Shivani R Babu [2], Robert L Judd [2,*] and Thangiah Geetha [1,*]

[1] Department of Nutritional Sciences, Auburn University, Auburn, AL 36849, USA;
[2] Department of Anatomy, Physiology and Pharmacology, Auburn University, Auburn, AL 36849, USA
* Correspondence: juddrob@auburn.edu (R.L.J.); thangge@auburn.edu (T.G.);
Tel.: +1-(334)-844-5416 (R.L.J.); +1-(334)-844-7418 (T.G.)

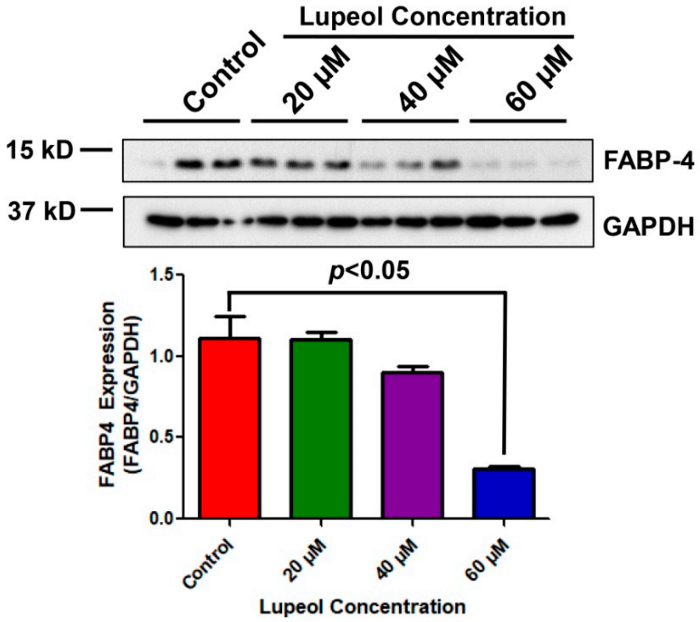

Figure S1. Dose dependent effect of lupeol on the expression of FABP4 by western blot analysis.
