# Supplementary figures and images for: Lupeol Attenuates Palmitate-Induced Hypertrophy in 3T3-L1 Adipocytes
Source: Biomolecules. 2025 Jan 15;15(1):129. doi: 10.3390/biom15010129 (PMC11763665; doi:10.3390/biom15010129)

Figure 1c

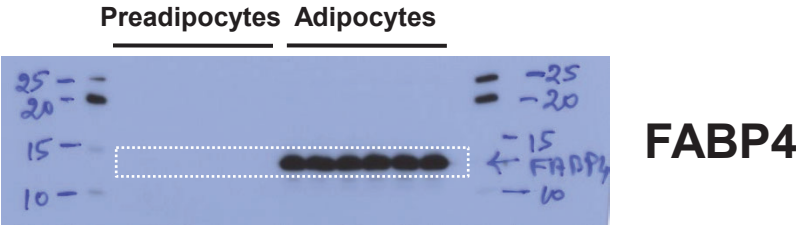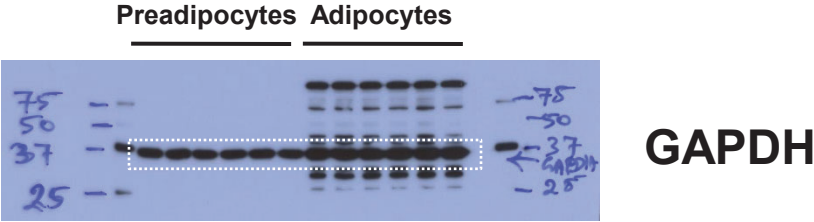

Figure 5

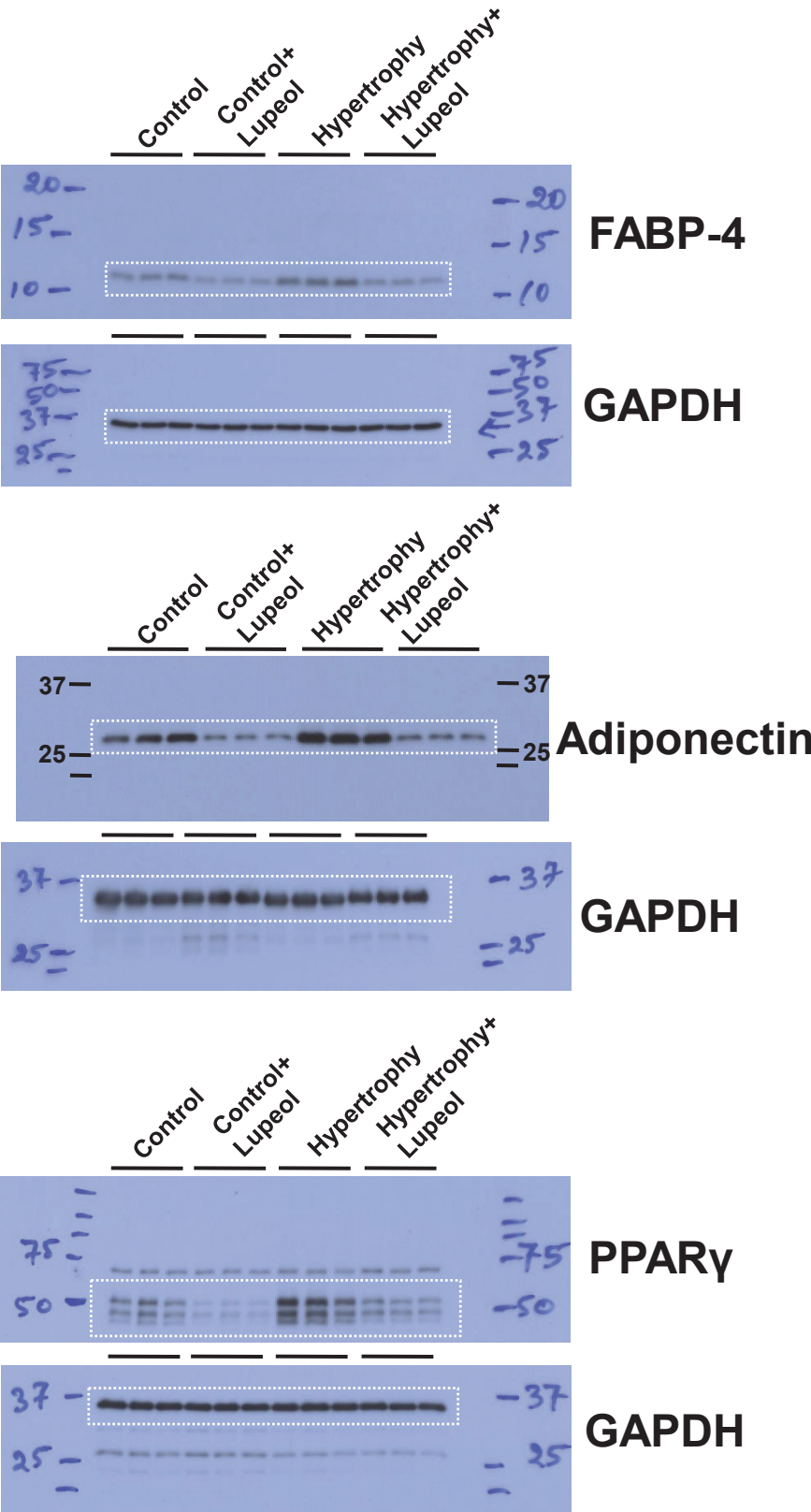

Figure 6

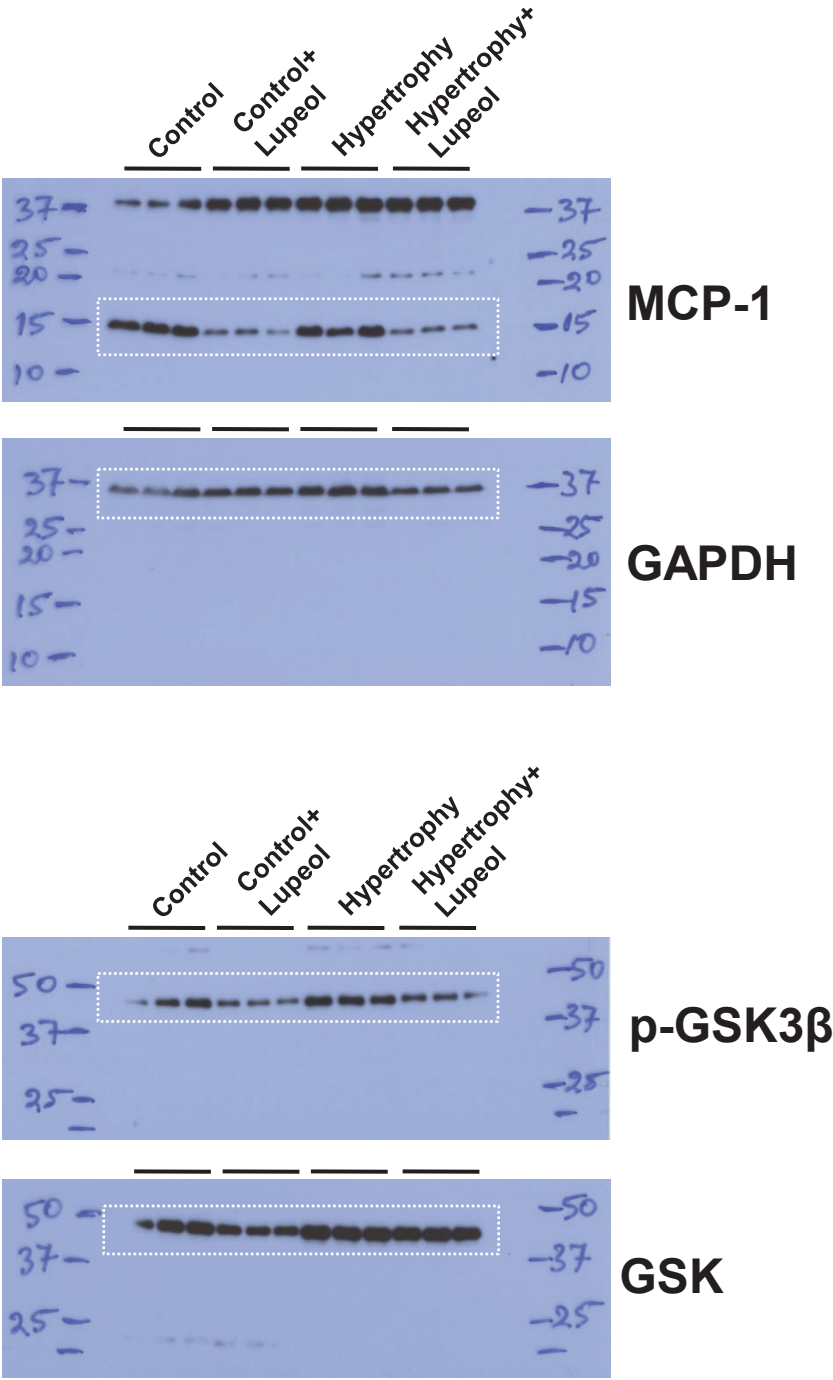

### Figure 7

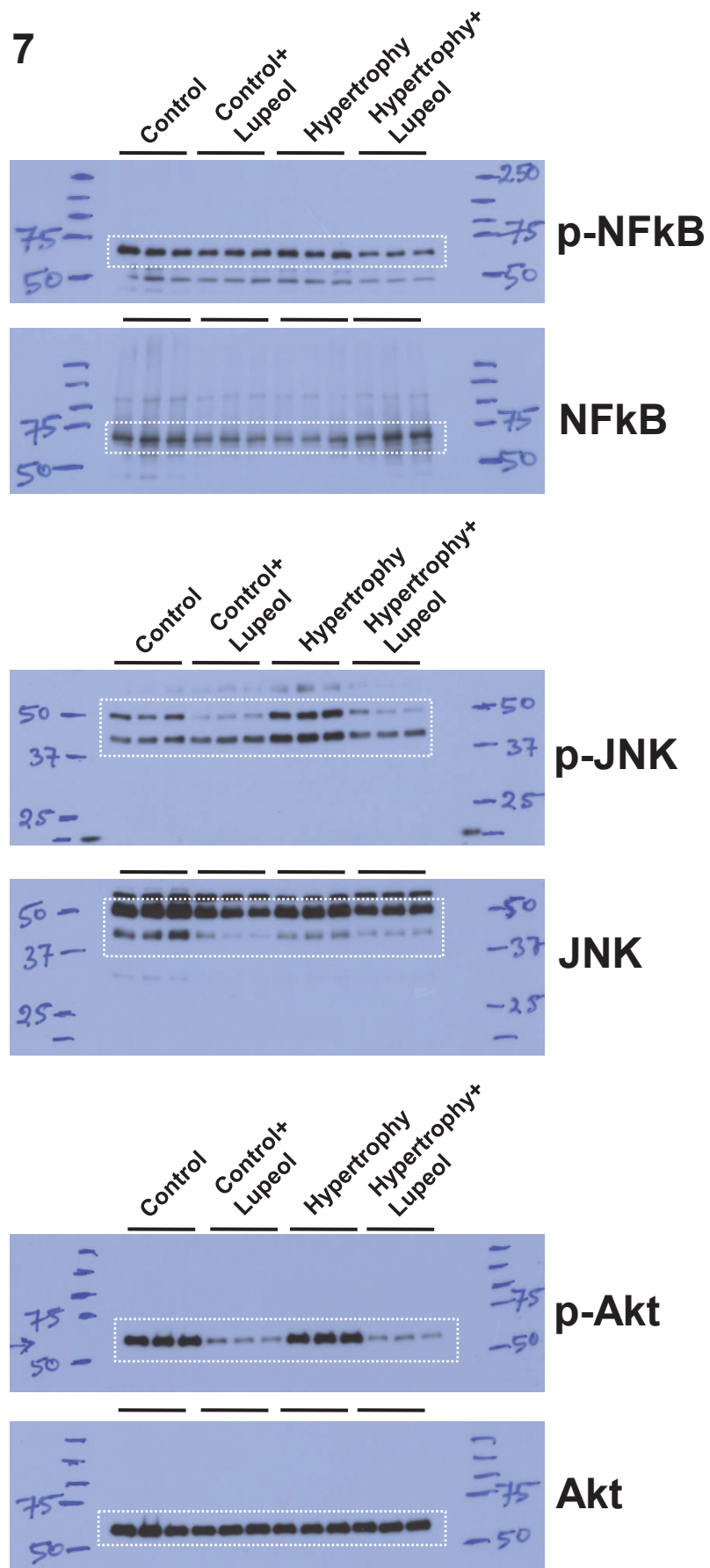

Figure 8

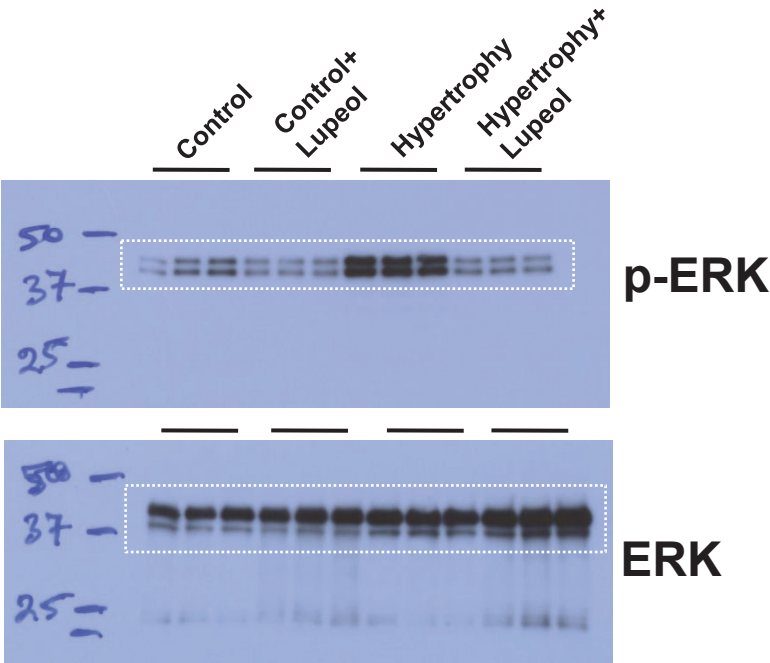

Supplement: Supplementary file 1 [file biomolecules-15-00129-s001.zip › biomolecules-3376086-original-images.pdf]
